# Supplementary material for: The queenslandensis and the type Form of the Dengue Fever Mosquito (Aedes aegypti L.) Are Genomically Indistinguishable
Source: PLoS Negl Trop Dis. 2016 Nov 2;10(11):e0005096. doi: 10.1371/journal.pntd.0005096 (PMC5091912; doi:10.1371/journal.pntd.0005096)
Supplement: S1 Table — Mosquitoes from Rio de Janeiro (Brazil), Gordonvale (northern Queensland), Ho Chi Minh city (Vietnam), used in the DAPC analysis. (PDF) [file pntd.0005096.s001.pdf]

S1 Table.

sample_ID	region	lat	lon	sampling method	life stage	sex	form	het loci	depth	miss loci
QLD_G_01-01	Gordonvale, QLD	-17.08808	145.78595	BG-Sentinel trap	adult	female	*type*	0.20	6.8	0.264
QLD_G_05-01	Gordonvale, QLD	-17.09640	145.78479	BG-Sentinel trap	adult	female	*type*	0.19	9.3	0.167
QLD_G_06-02	Gordonvale, QLD	-17.08697	145.78795	BG-Sentinel trap	adult	female	*type*	0.20	8.2	0.200
QLD_G_08-01	Gordonvale, QLD	-17.08805	145.78708	BG-Sentinel trap	adult	female	*type*	0.23	12.9	0.061
QLD_G_08-04	Gordonvale, QLD	-17.08805	145.78708	BG-Sentinel trap	adult	female	*type*	0.18	10.5	0.112
QLD_G_11-01	Gordonvale, QLD	-17.09820	145.78293	BG-Sentinel trap	adult	female	*type*	0.21	9.2	0.154
QLD_G_11-03	Gordonvale, QLD	-17.09820	145.78293	BG-Sentinel trap	adult	female	*type*	0.21	7.7	0.242
QLD_G_12-01-01	Gordonvale, QLD	-17.09586	145.78059	BG-Sentinel trap	adult	female	*type*	0.15	5.6	0.359
QLD_G_22-11	Gordonvale, QLD	-17.08923	145.78517	BG-Sentinel trap	adult	female	*type*	0.21	10.7	0.108
QLD_G_22-19	Gordonvale, QLD	-17.08923	145.78517	BG-Sentinel trap	adult	female	*type*	0.21	12.0	0.077
QLD_G_23-04	Gordonvale, QLD	-17.09008	145.78402	BG-Sentinel trap	adult	female	*type*	0.18	11.7	0.100
QLD_G_26-02	Gordonvale, QLD	-17.09431	145.78465	BG-Sentinel trap	adult	female	*type*	0.22	11.8	0.085
QLD_G_26-15	Gordonvale, QLD	-17.09431	145.78465	BG-Sentinel trap	adult	female	*type*	0.20	11.3	0.084
QLD_G_28-01	Gordonvale, QLD	-17.09909	145.77921	BG-Sentinel trap	adult	female	*type*	0.19	7.8	0.226
QLD_G_92-01	Gordonvale, QLD	-17.09333	145.78364	BG-Sentinel trap	adult	female	*type*	0.19	7.4	0.241
BrK11-01-1	Tubiacanga, Rio de Janeiro	-22.78507	-43.22711	ovitrap	larva	female	-	0.19	11.4	0.114
BrK11-06-2	Tubiacanga, Rio de Janeiro	-22.78439	-43.22669	ovitrap	larva	male	-	0.18	8.8	0.192
BrK11-09-4	Tubiacanga, Rio de Janeiro	-22.78489	-43.22501	ovitrap	larva	male	-	0.20	9.3	0.177
BrK11-13-1	Tubiacanga, Rio de Janeiro	-22.78523	-43.22512	ovitrap	larva	female	-	0.19	10.7	0.127
BrK11-18-1	Tubiacanga, Rio de Janeiro	-22.78551	-43.22561	ovitrap	larva	male	-	0.18	10.4	0.144
BrK11-21-3	Tubiacanga, Rio de Janeiro	-22.78578	-43.22522	ovitrap	larva	female	-	0.19	10.2	0.143
BrK11-23-1	Tubiacanga, Rio de Janeiro	-	-	ovitrap	larva	male	-	0.21	14.1	0.069
BrK11-24-2	Tubiacanga, Rio de Janeiro	-22.78655	-43.22656	ovitrap	larva	male	-	0.21	13.3	0.075
BrK11-25-2	Tubiacanga, Rio de Janeiro	-22.78450	-43.22653	ovitrap	larva	male	-	0.15	12.8	0.097
BrK11-26-1	Tubiacanga, Rio de Janeiro	-22.78561	-43.22601	ovitrap	larva	male	-	0.20	10.4	0.145
BrK11-27-1	Tubiacanga, Rio de Janeiro	-22.78559	-43.22659	ovitrap	larva	male	-	0.21	13.3	0.076
BrK11-45-1	Tubiacanga, Rio de Janeiro	-22.78517	-43.22684	ovitrap	larva	female	-	0.20	10.8	0.133
BrK11-53-1	Tubiacanga, Rio de Janeiro	-22.78455	-43.22583	ovitrap	larva	male	-	0.18	10.3	0.154
BrK11-54-2	Tubiacanga, Rio de Janeiro	-22.78435	-43.22655	ovitrap	larva	male	-	0.19	8.5	0.202
BrK11-60-1	Tubiacanga, Rio de Janeiro	-22.78426	-43.22812	ovitrap	larva	male	-	0.20	10.0	0.171
Vi14-01-wt	Ho Chi Minh City	-	-	ovitrap	larva	male	-	0.18	7.2	0.252
Vi14-02-wt	Ho Chi Minh City	-	-	ovitrap	larva	male	-	0.20	9.8	0.124
Vi14-03-wt	Ho Chi Minh City	-	-	ovitrap	larva	male	-	0.19	6.7	0.283

Vi14-04-wt	Ho Chi Minh City	-	-	ovitrap	larva	male	-	0.21	10.1	0.114
Vi14-05-wt	Ho Chi Minh City	-	-	ovitrap	larva	male	-	0.19	7.5	0.227
Vi14-06-wt	Ho Chi Minh City	-	-	ovitrap	larva	male	-	0.19	8.1	0.195
Vi14-07-wt	Ho Chi Minh City	-	-	ovitrap	larva	male	-	0.09	7.4	0.221
Vi14-10-wt	Ho Chi Minh City	-	-	ovitrap	larva	female	-	0.19	8.0	0.213
Vi14-11-wt	Ho Chi Minh City	-	-	ovitrap	larva	female	-	0.15	6.4	0.294
Vi14-12-wt	Ho Chi Minh City	-	-	ovitrap	larva	male	-	0.18	6.1	0.314
Vi14-14-wt	Ho Chi Minh City	-	-	ovitrap	larva	female	-	0.14	7.4	0.250
Vi14-16-wt	Ho Chi Minh City	-	-	ovitrap	larva	female	-	0.17	6.5	0.296
Vi14-17-wt	Ho Chi Minh City	-	-	ovitrap	larva	male	-	0.18	7.1	0.234
Vi14-18-wt	Ho Chi Minh City	-	-	ovitrap	larva	female	-	0.15	5.5	0.378
Vi14-19-wt	Ho Chi Minh City	-	-	ovitrap	larva	male	-	0.19	7.2	0.254
