# Supplementary figures and images for: The queenslandensis and the type Form of the Dengue Fever Mosquito (Aedes aegypti L.) Are Genomically Indistinguishable
Source: PLoS Negl Trop Dis. 2016 Nov 2;10(11):e0005096. doi: 10.1371/journal.pntd.0005096 (PMC5091912; doi:10.1371/journal.pntd.0005096)

## Singapore

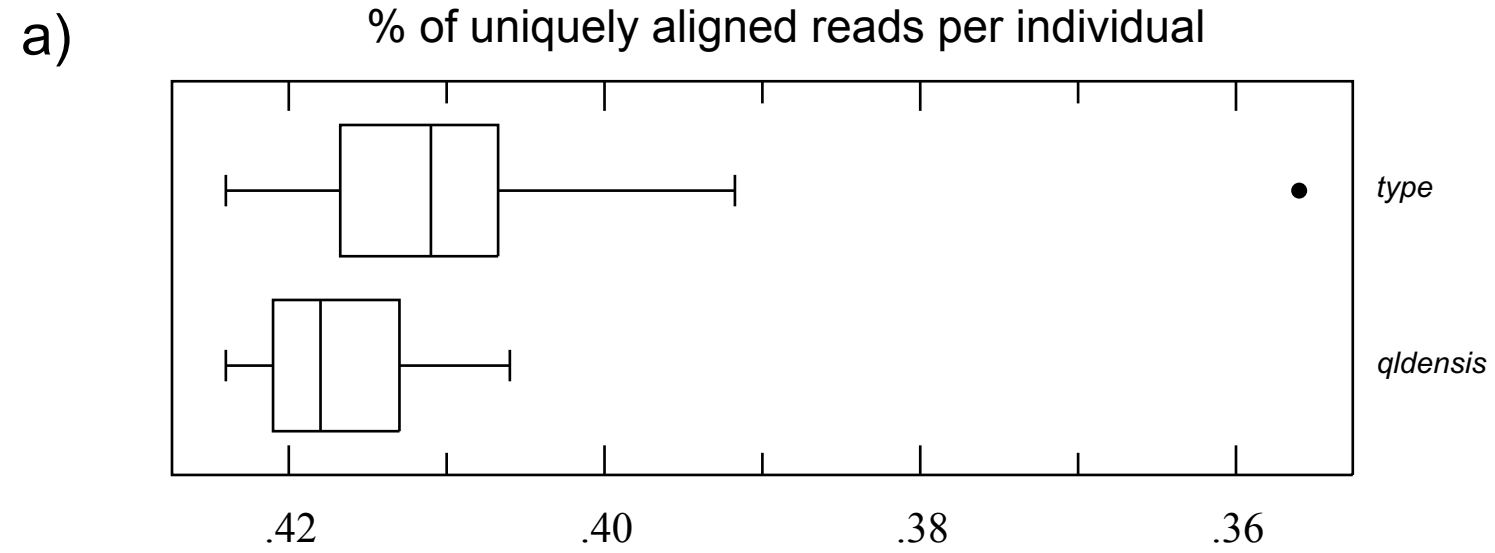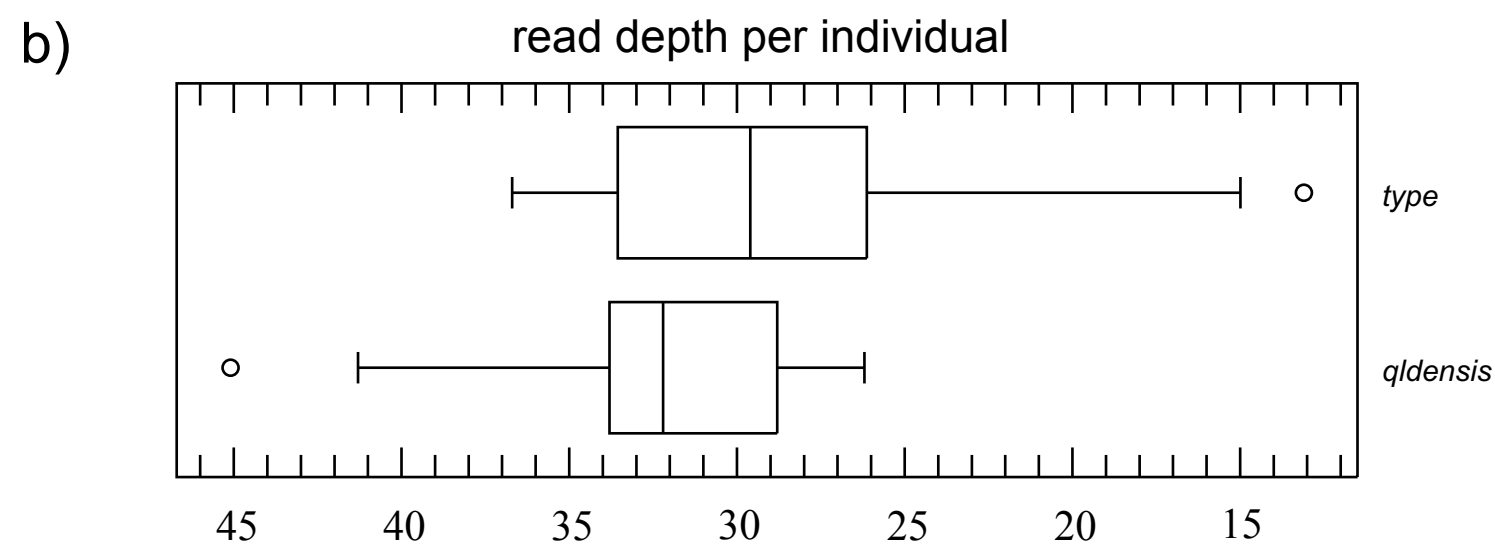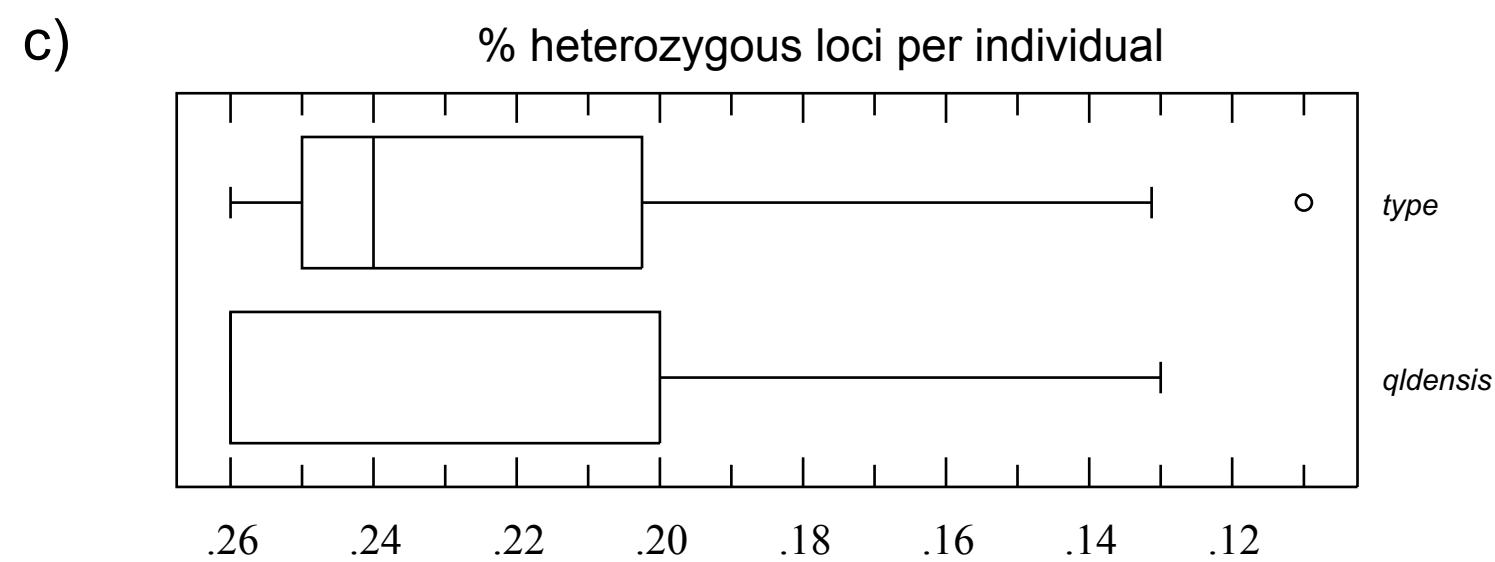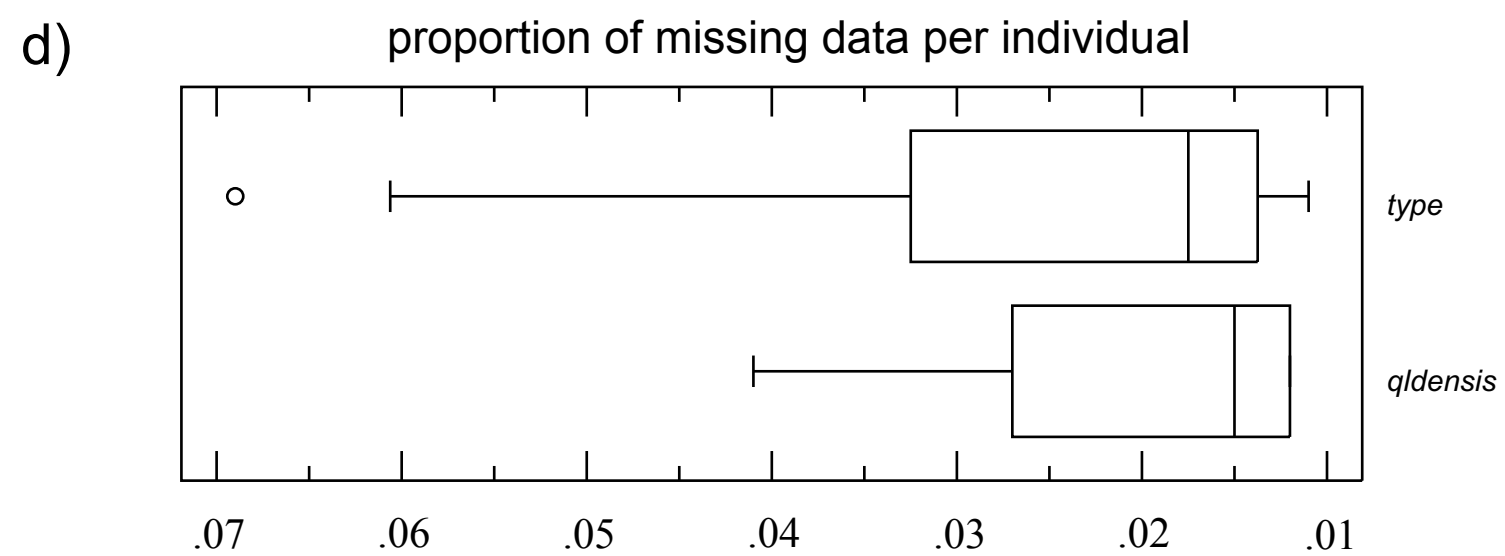

## Queensland

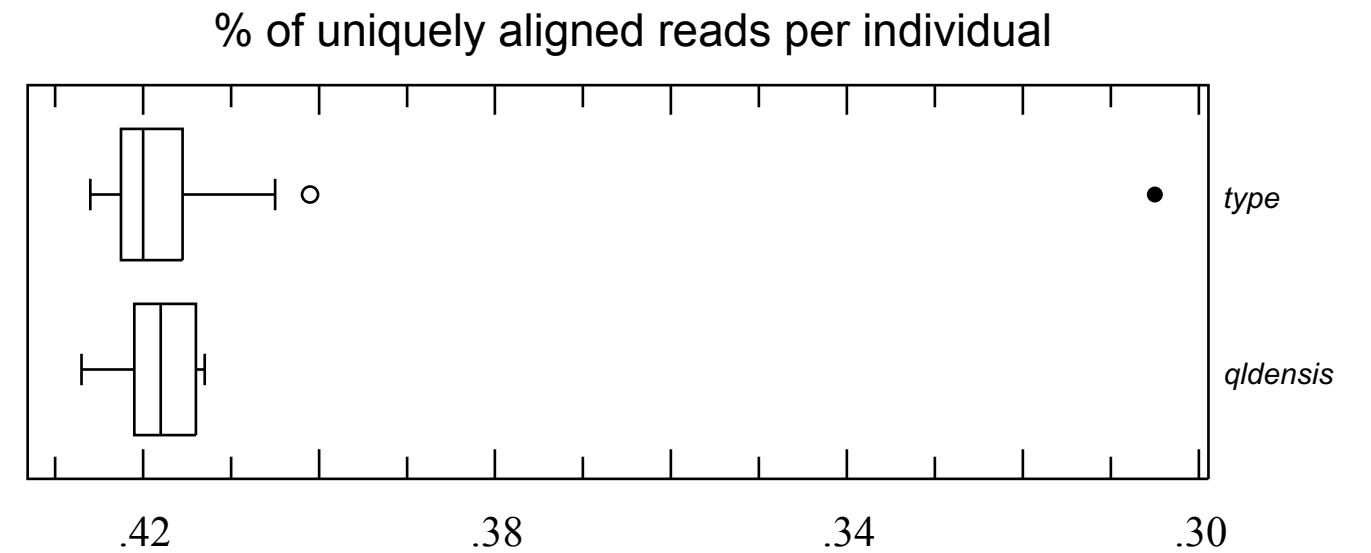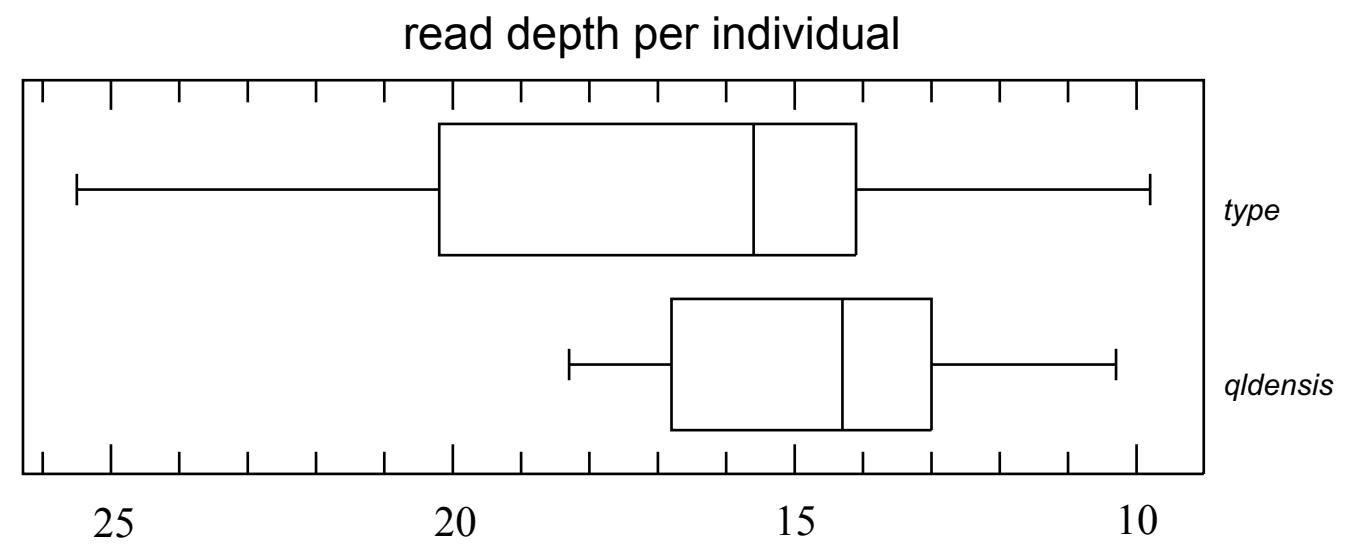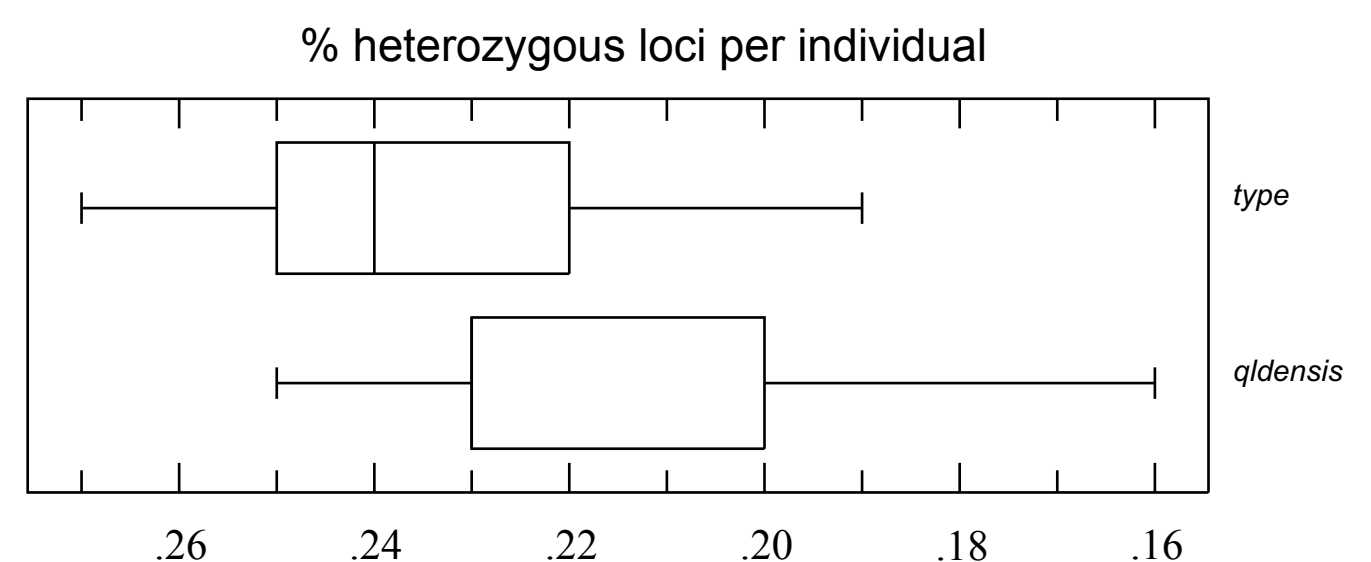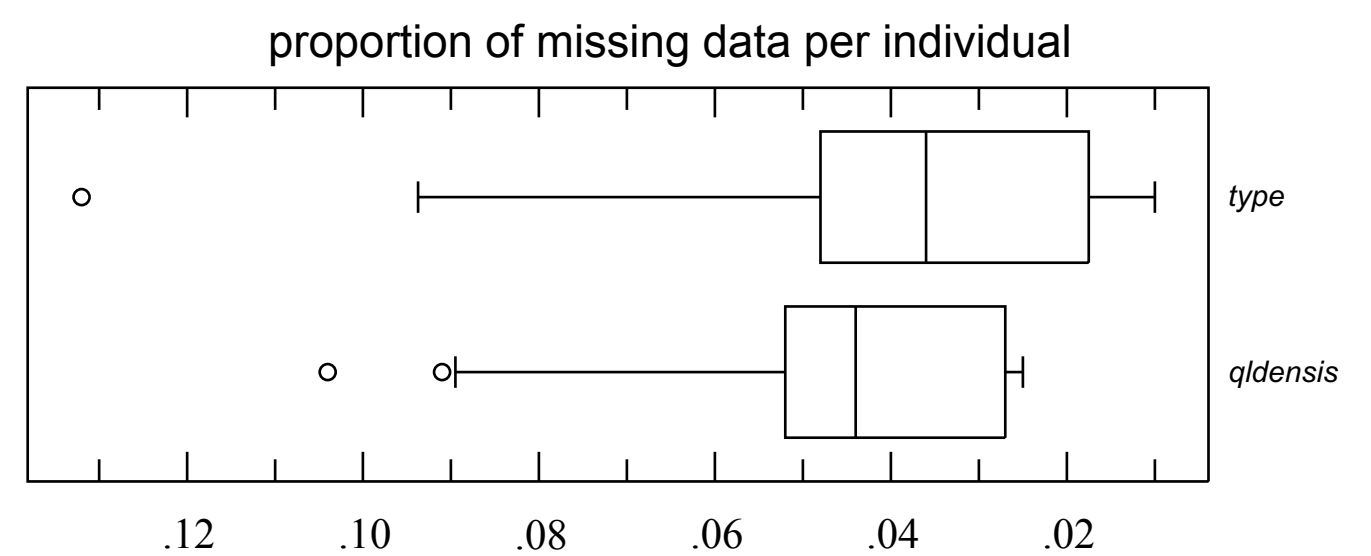

Supplement: S1 Fig — Boxplots of per-individual values for the proportion of uniquely aligned reads, RAD tag read depth, proportion of heterozygous loci, proportion of missing data for Aedes aegypti from Singapore (left) and Queensland (right). (PDF) [file pntd.0005096.s005.pdf]
